# Black Hole Quencher‐Enhanced Plasmonic Photothermal Conversion

**DOI:** 10.1002/advs.76680

**Published:** 2026-07-14

**Authors:** Ruiyuan Zhang, Xinru Chen, Lin Shen, Jin Li

**Affiliations:** ^1^ School of Information and Electronic Engineering Shandong Technology and Business University Yantai China; ^2^ CAS Key Laboratory of Coastal Environmental Processes and Ecological Remediation, Yantai Institute of Coastal Zone Research Chinese Academy of Sciences Yantai China; ^3^ Faculty of Biomedical Engineering Shenzhen University of Advanced Technology Shenzhen China

**Keywords:** black hole quencher, electromagnetic field, photothermal therapy, plasmonic photothermal conversion, thermoplasmonics

## Abstract

A common goal in the field of thermoplasmonics is to improve the photothermal conversion efficiency (PCE) of plasmonic nanostructures, yet it is highly challenging. We herein report a universal method to dramatically improve plasmonic photothermal conversion by add‐on black hole quencher (BHQ) molecules. BHQs, nonfluorescent resonant molecules, are immobilized to matched metal nanostructures to afford the excited coupling plasmonic hybrids with charge/energy transfer upon laser irradiation. Photothermal efficacy test confirms that the PCE of the hybrids is much higher than that of the sum of single plasmon and BHQ alone, leading to enhanced plasmonic photothermal conversion by approximately three times. Moreover, the PCE of the hybrid nanoparticles exhibits a nonlinear response to the plasmon fields, a phenomenon that cannot be achieved by fluorophores. The comparative experiments suggest that the whole photothermal efficacy of this hybrid stems from plasmonic nanoparticles, BHQ itself, as well as the mutual charge/energy transfer interaction. We demonstrate the generalizability of the strategy on various plasmonic nanostructures with three absorption‐matched BHQs. The resultant BHQ‐plasmon hybrids with enhanced conversion as high as 69.1% in NIR‐II, which allows for highly efficient hyperthermia ablation of cancer cells in vitro and deep‐seated tumors in vivo.

## Graphical abstract

Researchers have developed a universal method to boost plasmonic photothermal conversion by attaching black hole quencher (BHQ) molecules to matched metal nanostructures. This hybrid approach increases efficiency by roughly threefold, reaching up to 69.1% in the near‐infrared‐II window. The enhanced performance enabled effective hyperthermia destruction of cancer cells in vitro and deep tumors in vivo.

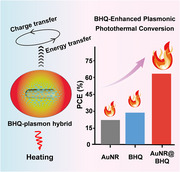

## 1 Introduction

Over the past two decades, plasmonic nanoparticles (NPs) have drawn increasing attention as remotely controllable heat sources driven by light, which has led to the emergence of thermoplasmonics as a field [1, 2, 3]. Their great potential spans a wide array of applications, including photothermal therapy (PTT), photothermal chemistry [4, 5], photothermal cell biology [6], photothermal polymerase chain reaction [7], and immunosorbent assay [8], solar light harvesting [9], and thermoplasmonic ignition [10], and so on (Figure S1). Across all these thermoplasmonic applications, a shared goal is to maximize the light‐to‐heat performance of plasmonic nanostructures, i.e., their photothermal conversion efficiency (PCE). Recent decades have seen many experimental studies aimed at improving the PCE of plasmonic nanostructures, such as tuning the size [11], morphology [12], and composition [13]. Besides, surface hybridization of the native organic molecules with plasmon has also been proven to be highly effective. For example, cobalt porphyrin adsorbed on plasmonic gold NPs (AuNPs) leads to enhanced hot carrier and local heating generation for efficient photocatalysis [14]. Photoconductive polymer phthalocyanine coatings coated onto Au nanorods (AuNRs) result in resonance energy transfer, and the resultant hybrid nanoantennas achieve ca. 50% efficiency of non‐radiative energy transfer [15]. Modification of AuNRs with carboxylate pillararene and photosensitizer leads to multifunctional hybrid nanotheranostic agents for photothermal‐photodynamic combination therapy [16]. However, developing a universal method to significantly boost the PCE of plasmonic nanostructures remains challenging.

Organic small molecules are attractive photothermal materials due to their flexibility, diversity, and tunable properties [17]. Many of them exhibit high molar absorption coefficients in specific spectral regions, enabling efficient light‐to‐heat conversion at targeted wavelengths and thus enhancing photothermal harvesting. Black hole quencher (BHQ) molecules are another class of nonfluorescent resonant molecules [18, 19, 20], which have drawn much attention due to their strong resonant absorption and satisfactory photothermal conversion efficiency. Jablonski diagram illustrates that the transition of electrons within the molecule from the excited state back to the ground state through non‐radiative pathways (such as thermal vibrations), rather than emitting photons via radiative pathways such as fluorescence. Thus, surface modification of plasmonic nanostructures with BHQ molecules is expected to yield favorable photothermal agents with several unique advantages: (1) absorption of BHQ molecules and plasmonic nanostructures could be tuned to match, thereby improving light and heat harvesting [21]; (2) non‐radiative decay of the excited BHQ molecules may be further maximized owing to the plasmonic effect [22, 23]; (3) photobleaching of the hybrids could be overcome due to the fluorescence‐free features of BHQs [24, 25]. Therefore, rationally combining BHQs with plasmonic fields offers an effective strategy to obtain hybrid plasmonic nanomaterials with enhanced photothermal performance and photostability.

**SCHEME 1:**
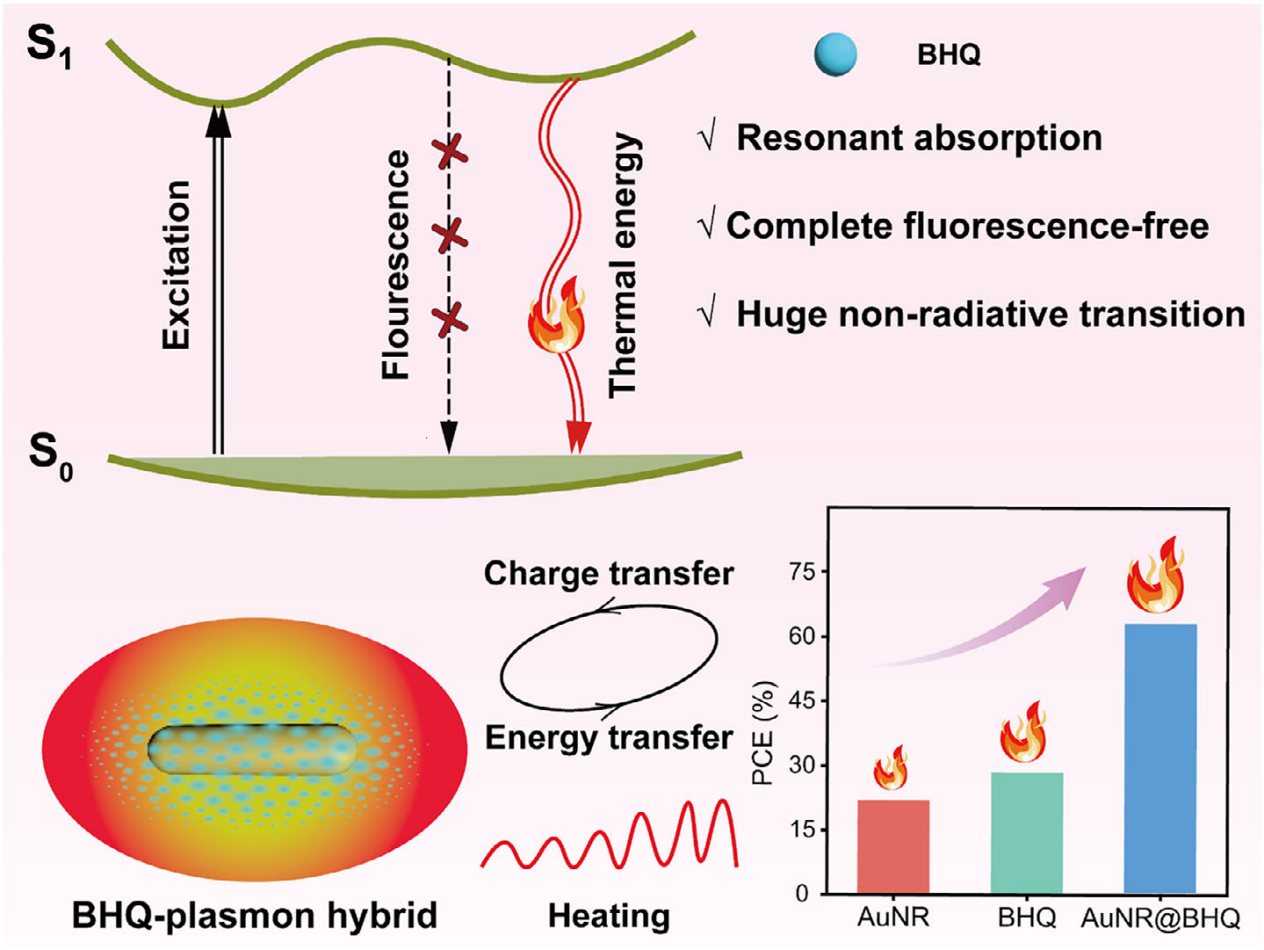
Schematic diagram of BHQs and BHQ‐enhanced plasmonic photothermal conversion.

Inspired by this, we here report a universal method to boost plasmonic photothermal conversion by a combination of BHQ molecules and plasmon nanostructures. BHQs, nonfluorescent resonant molecules, are immobilized on the surface of matched plasmon nanostructures to afford the excited coupling plasmonic hybrids upon laser irradiation. Photothermal efficacy test reveals that the PCE of the hybrids is much higher than that of the sum of normal plasmon nanostructures and BHQ alone (Scheme 1), leading to enhanced plasmonic photothermal conversion by approximately three times. Through various comparative experiments, we attribute this enhanced photothermal effect to the electromagnetic field that amplifies the non‐radiative transitions of BHQ molecules. Moreover, PCE exhibits a nonlinear response to the local field of plasmons, which cannot be realized by fluorescent molecules. We demonstrated the generalizability of the strategy on various plasmonic nanostructures with three absorption‐matched BHQs. Compared to pure plasmonic counterparts, the BHQ‐plasmon hybrids show far superior photothermal conversion and hyperthermia ablation in both cellular and murine tumor models, thereby confirming that our approach is viable for practical applications.

## 2 Results and Discussion

### 2.1 Photophysical Properties and Non‐Emissive Mechanism of BHQs

**FIGURE 1:**
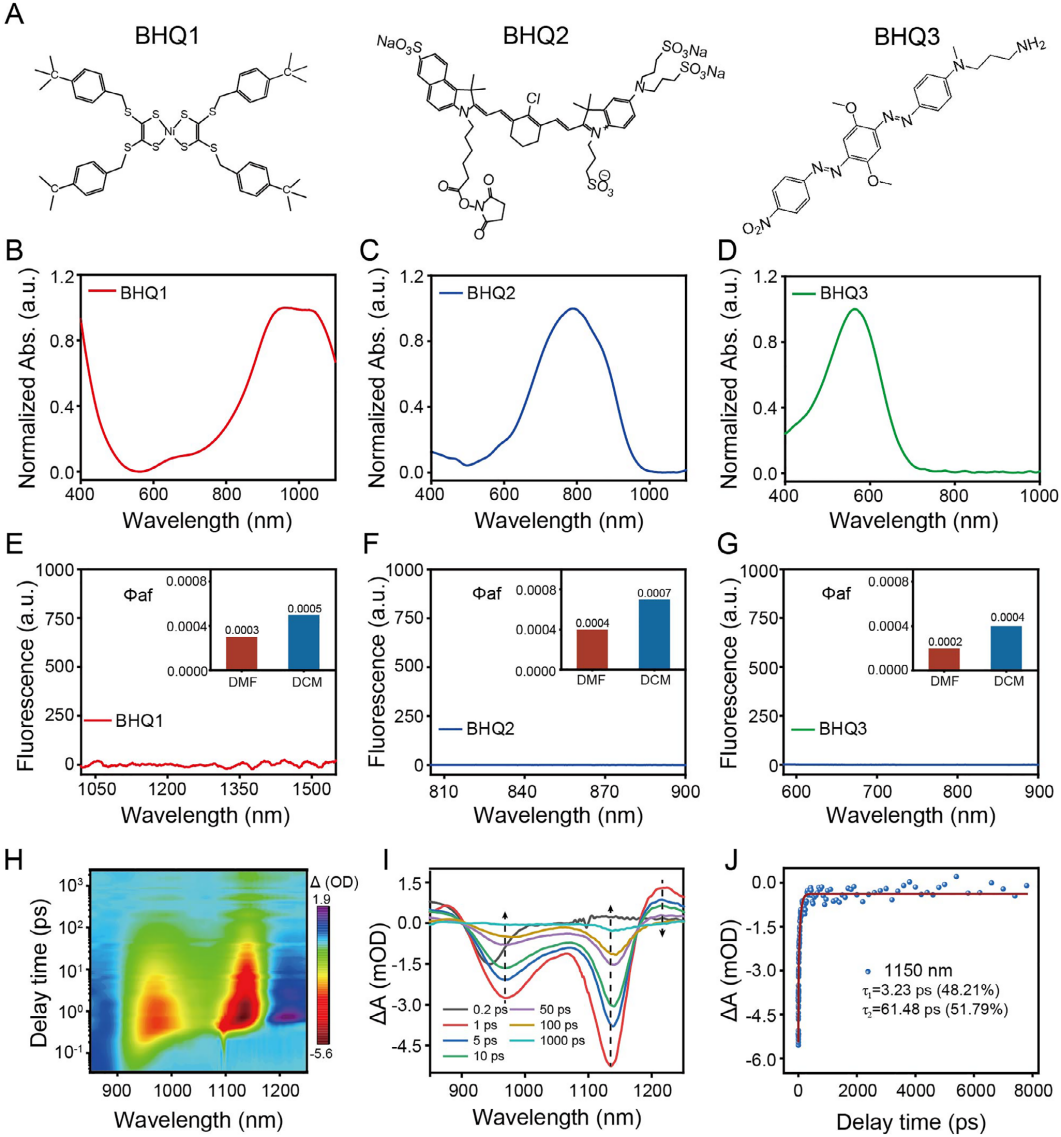
(A) Molecular structure of BHQ1, BHQ2, and BHQ3. (B) Normalized UV–vis–NIR spectra of BHQ1 in DMF. Normalized UV–vis spectra of (C) BHQ2 and (D) BHQ3 in DMF. (E–G) Fluorescence emission spectra of BHQ1, BHQ2 and BHQ3 excited by 1002, 805 and 584 nm, respectively. The insets show the AQY of these BHQs in DCM and DMF. (H) Pseudocolor fs‐TA mapping of BHQ1 in DMF excited by a 1000 nm pump laser. (I) fs‐TA plots of BHQ1 recorded from 0.2 to 1000 ps after laser pulse excitation. The arrows indicate the plot trends. (J) Kinetic traces and corresponding fitting lines within the GSB regime at 1150 nm.

Three BHQs are employed, and the molecular structures are shown in Figure 1A. BHQ1 and BHQ3 are homemade according to our previous works [18, 26], and BHQ2 is commercially available [27]. These BHQs are different from common fluorophores as controls employed in this work (Figure S2). Among them, BHQ1 molecules are nickel‐bis(dithiolene) organometallics in the second near‐infrared (NIR‐II) window [28, 29, 30]; BHQ2 molecules are cyanine derivatives in the first NIR (NIR‐I) window [31]; BHQ3 molecules are azobenzene compounds in the visible window [18]. These three molecules exhibit maximum absorption at 982, 785 and 564 nm, respectively (Figure 1B–D), supporting their strong photon absorption ability.

Next, we delved into the fluorescence emissions of these BHQs under laser excitation. At the same concentration of 10 µM, the emissions of the three BHQs in solutions are close to zero upon excitation at their maximum absorption peak of 1002, 805, and 584 nm, respectively. (Figure 1E–G). The absolute quantum yields (AQY) [32] of BHQ1 in N, N‐dimethylacetamide (DMF) and dichloromethane (DCM) are 0.0003 and 0.0005, respectively (the inset in Figure 1E), which is the lowest value for NIR‐II absorbing dyes to date [33, 34]. Such a low AQY originated from the narrow energy gap of the dyes and enhanced the non‐radiative transitions through internal conversion, consistent with the energy gap law [17]. Likewise, the AQY of BHQ2 and BHQ3 is almost negligible (the inset in Figure 1F,G). These results unambiguously reveal that three BHQs dissipate the excited energy through non‐radiative pathways rather than emitting fluorescence.

To delve into the underlying non‐emissive mechanism of BHQ1, we carried out ultrafast spectroscopy with sub‐picosecond time resolution by femtosecond transient absorption spectra (fs‐TA) [35, 36]. Two‐dimensional mapping shows positive and negative signals, attributed to excited‐state absorption and ground‐state bleaching (GSB), respectively (Figure 1H). In a one‐dimensional fs‐TA plot, the signals quickly decrease from 1 to 100 ps in the spectral region of 900–1150 nm and completely bleach within 1000 ps (Figure 1I), suggesting a rearrangement of the molecular conformation in excited states upon laser excitation. By fitting the band at 1150 nm in the GSB region with a double exponential function, we get that the two species have different component ratios and time constants (Figure 1J). The first fast time constant of 3.23 ps may correspond to the internal conversion from S_n_ to the first (S_1_), while the second time constant of 61.48 ps corresponds to the internal conversion from S_1_ to the ground state (S_0_). In short, these data uncover that the general nonfluorescent mechanism of BHQs could be ascribed to the ultrafast internal conversion, which may be further magnified by electromagnetic fields with plasmons to boost plasmonic photothermal conversion.

### 2.2 BHQ‐Enhanced Plasmonic Photothermal Conversion via Self‐Assembled Monolayers

**FIGURE 2:**
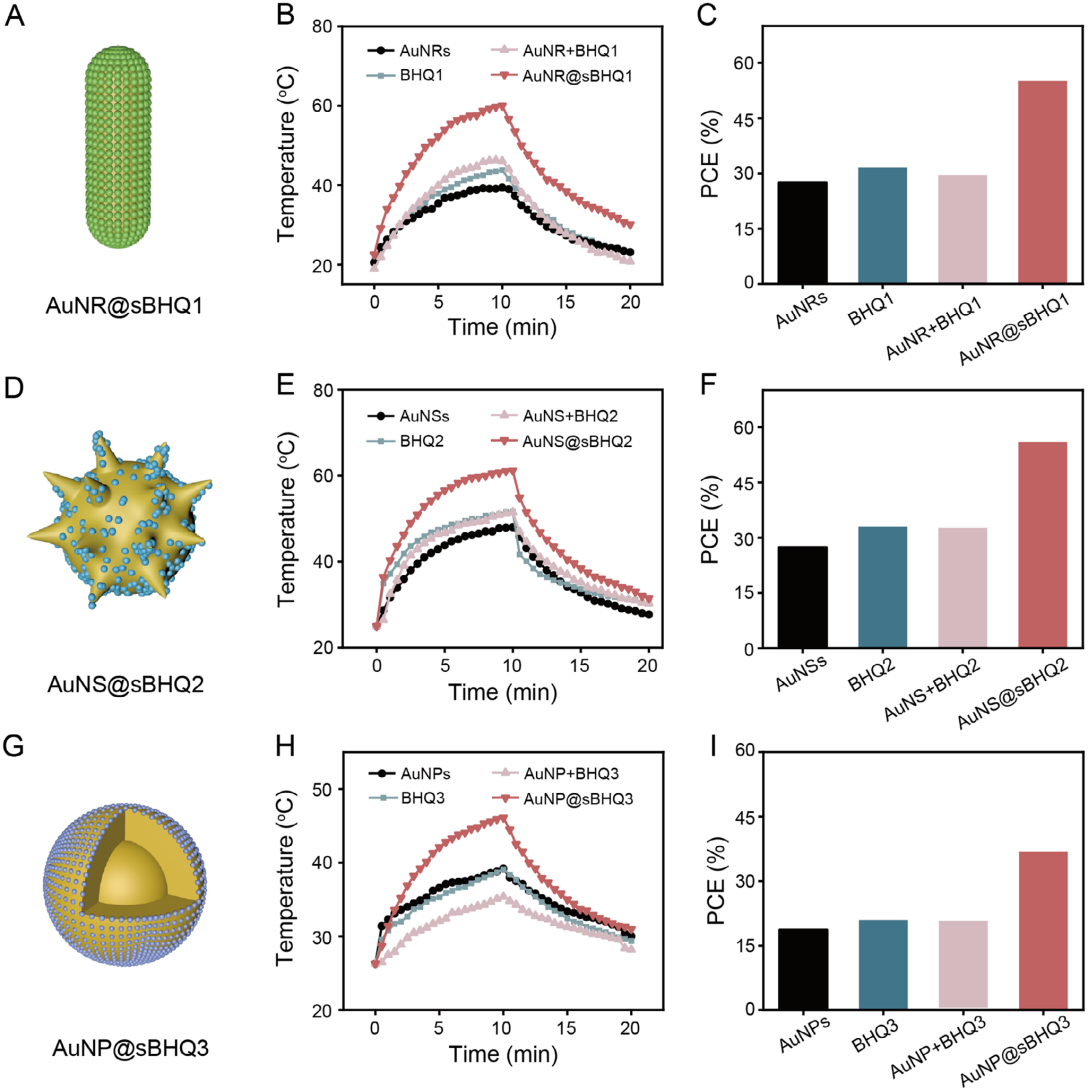
(A) Schematic diagram of AuNR@sBHQ1 nanostructure. (B) Heating–cooling curves of five groups with 1064 nm laser irradiation (1.0 W/cm^2^, 10 min) and (C) the corresponding PCE. (D) Schematic diagram of AuNS@sBHQ2 nanostructure. (E) Heating–cooling curves of five groups with 808 nm laser irradiation (1.0 W/cm^2^, 10 min) and (F) corresponding PCE. (G) Schematic diagram of AuNP@sBHQ3 nanostructure. (H) Heating–cooling curves of five groups with 633 nm laser irradiation (150 mW/cm^2^, 10 min) and (I) the corresponding PCE.

Next, we explored the BHQ‐enhanced plasmonic photothermal conversion after immobilization of these BHQs onto the surface of plasmon nanostructures with self‐assembly monolayers (SAMs). Matching the maximum absorption of BHQ1, BHQ2, and BHQ3, three plasmon nanostructures, including AuNRs, gold nanostars (AuNSs), and AuNPs with LSPR peaks of 1050, 693, and 552 nm, were selected for this purpose. Three BHQs were anchored onto the surface of the AuNRs via the direct Au─S bonds or add‐on linkers (Figure S3A). Particularly, BHQ1 was anchored onto the surface of the AuNRs via multiple Au─S bonds [26], producing AuNR@sBHQ1 NPs with SAMs of BHQ1 molecules (Figure 2A). UV–vis absorption and surface‐enhanced Raman scattering (SERS) spectra collectively confirmed the BHQ molecule modification onto the plasmonic surface (Figure S3B,C). For example, the featured band at 1154 cm^−1^ in the Raman spectrum of the AuNR‐based hybrid NPs is assigned to the ν(C═C) from BHQ1 [26, 27], implying the successful modification of BHQ1 onto the AuNRs surface.

After successful preparation of these BHQ‐decorated hybrids, we studied the photothermal conversion capacity of AuNR@sBHQ1 NPs. Due to the highly inhibited fluorescence radiation transition, the photothermal properties of BHQ1 were first investigated. BHQ1 exhibits a high PCE of 31.5% (Figure 2B, blue line), which is higher than that of normal AuNRs (27.5%, gray line). This is something that most fluorophores are unable to achieve, such as common cyanine dyes [37], because of the giant radiative transitions that determine the relatively weak PCE. After that, we tested the PCE of AuNR@sBHQ1 NPs. Figure 2B shows the temperature‐cooling curves of different species under a 1064 nm laser with 1 W/cm^2^ power. The temperature of the hybrid NPs rapidly rises from 22.5°C to 60°C within 10 min, displaying the best temperature response among the three examined groups, including AuNRs and BHQ1. When the dye was assembled onto the surface of AuNRs, the resulting NPs showed a PCE of 55% (Figure 2C), greater than the sum of BHQ1 and AuNRs alone, indicating that BHQ1 plays a key role in the enhancement of PCE. (Figure 2C). This remarkable property with the hybrids implies the presence of a strong coupling between AuNRs and BHQ1 molecules. To explain the enhanced PCE, we assume that the plasmonic field significantly accelerates the non‐radiative transition process of non‐fluorescent molecules under laser irradiation from the excited state to the ground state as heat energy. To verify this, we simply mixed BHQ1 and AuNRs with a ratio of 11000, a value similar to that in AuNR@sBHQ1 NPs, and measured the PCE of the sample (BHQ1+AuNRs) soon. In this case, the plasmonic field surrounding AuNRs exerts negligible influence on BHQ1. It is found that the PCE is only 29.5%, far lower than AuNR@sBHQ1 NPs (55%). This controlled experiment rationalizes our speculation that the accelerated non‐radiative transition, as heating in hybrid AuNR@sBHQ1 NPs, is indeed caused by the plasmonic field [22].

Likewise, we prepared BHQ2‐anchored AuNSs with SAMs (AuNS@sBHQ2 NPs) by chemically adsorbing them onto the surface of AuNSs (Figure 2D, see supporting information for more details). For this, AuNSs were first conjugated to mercaptoethylamine, followed by reacting with IQ1 between NHS and NH_2_. Under 808 nm laser illumination, we measured the photothermal cooling curve of the hybrid NPs (Figure 2E) and calculated the PCE according to the cooling function. As expected, AuNS@BHQ2 NPs exhibit a remarkable PCE (55.8%), surpassing that of AuNSs (27.4%) and the molecules (32.9%) (Figure 2F). Such a high conversion efficiency does not result from a simple mixture of the two, as evidenced by the fact that the PCE from the mixture is only 35% (Figure 2F, yellow line). Also, we prepared BHQ3‐decorated AuNPs NPs (Figure 2G, see supporting information for more details). To this end, AuNPs were first conjugated to mercaptoacetic acid, followed by reacting with BHQ3 between COOH and NH_2_ through EDC/NHS chemistry, producing AuNP@sBHQ3 NPs. Under 633 nm laser illumination, we measured the photothermal cooling curve of the hybrid NPs (Figure 2H) and calculated the PCE as high as 36.7%, greater than that of AuNPs (18.7%) as well as BHQ3 (20.9%) (Figure 2I). All the above outcomes clearly demonstrate BHQ‐enhanced plasmonic photothermal conversion regardless of the morphology and size.

### 2.3 Charge/Energy Transfer Mechanism at the BHQ/Au Interface

**FIGURE 3:**
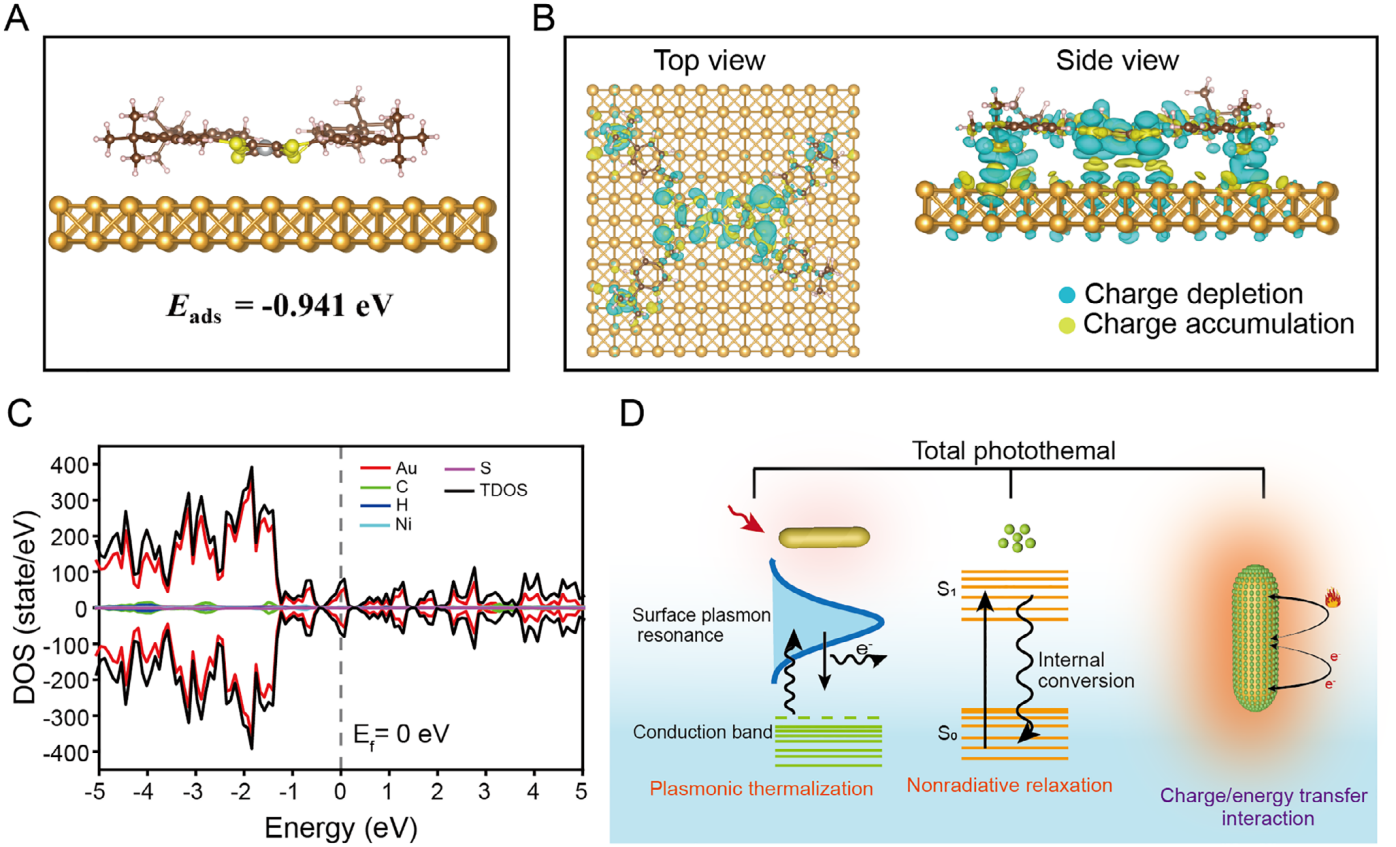
(A) Density Functional Theory Calculation of Adsorption Energy of BHQ1 on Au(111) Surface. (B) Differential charge densities of BHQ1 onto AuNPs. Yellow regions signify increased electron density, while blue regions indicate decreased electron density. (C) Density of states of Au@BHQ1 NPs. (D) Proposed photothermal mechanism for the BHQ‐modified plasmonic nanostructures.

To delve into the underlying charge/energy transfer mechanism between BHQ molecules and plasmons that accounts for the enhanced photothermal conversion of the hybrid, we performed density functional theory (DFT) calculations (see methods for details). First, adsorption energy was calculated. In Figure 3A, the adsorption energy of BHQ1 on the Au(111) surface was calculated to be −0.941 eV. This large negative value indicates the formation of stable adsorption conformations for strong interaction [38]. To illustrate the interfacial charge transfer behavior, differential charge density analysis was further performed and shown in Figure 3B with both top and side views, where regions of electron depletion are colored blue, and regions of electron accumulation are colored yellow [39]. This reveals that electrons migrate from the Au(111) surface to the heteroatomic sites of BHQ1 molecules. Furthermore, density of states (DOS) [40, 41] analysis shows that the electronic states of Au are significantly distributed in the energy range of −5 to 5 eV. Although there is a distinct difference between Au and BHQ1 in the DOS in the range of −5 to −2 eV, the 5d/6s orbitals of Au extensively overlap with the HOMO/LUMO orbitals of BHQ1 in the energy range of −2 to 0 eV (Figure 3C), confirming the existence of strongly electronic orbital hybridization between the two in this interval [39]. Correspondingly, the energy level data indicate that the band gap of BHQ1 molecules sharply shrinks from 0.6138 to 0.0249 eV after adsorption to Au (Table S1), which illustrates significant charge redistribution at the BHQ1/Au(111) interface in the chemical adsorption model for energy transfer [14, 42]. Overall, these calculations indicate that the strong charge/energy transfer between BHQ and Au accelerates thermalization by promoting non‐radiative decay (internal conversion and vibrational dissipation), rather than relying solely on spectral overlap, thus explaining the enhanced photothermal phenomenon after recombination [43, 44].

Previous works on improving plasmonic photothermal efficacy have focused only on the plasmon itself, such as defect damping engineering [45] or the coupling via mode hybridization [46, 47]. To the best of my knowledge, this is the first report of an additional BHQ‐enhanced plasmon photothermal effect by strong charge/energy transfer. These results lead us to propose a simple, new method for enhancing the photothermal efficiency of plasmonic nanomaterials. The whole photothermal efficacy stems from three integrated factors: (1) AuNRs via electron‐hole recombination, (2) BHQ1 itself via internal conversion, and (3) the enhancement part arising from their mutual interaction through charge/energy transfer (Figure 3D).

### 2.4 Maximizing Photothermal Conversion via Multilayer BHQ Encapsulation

**FIGURE 4:**
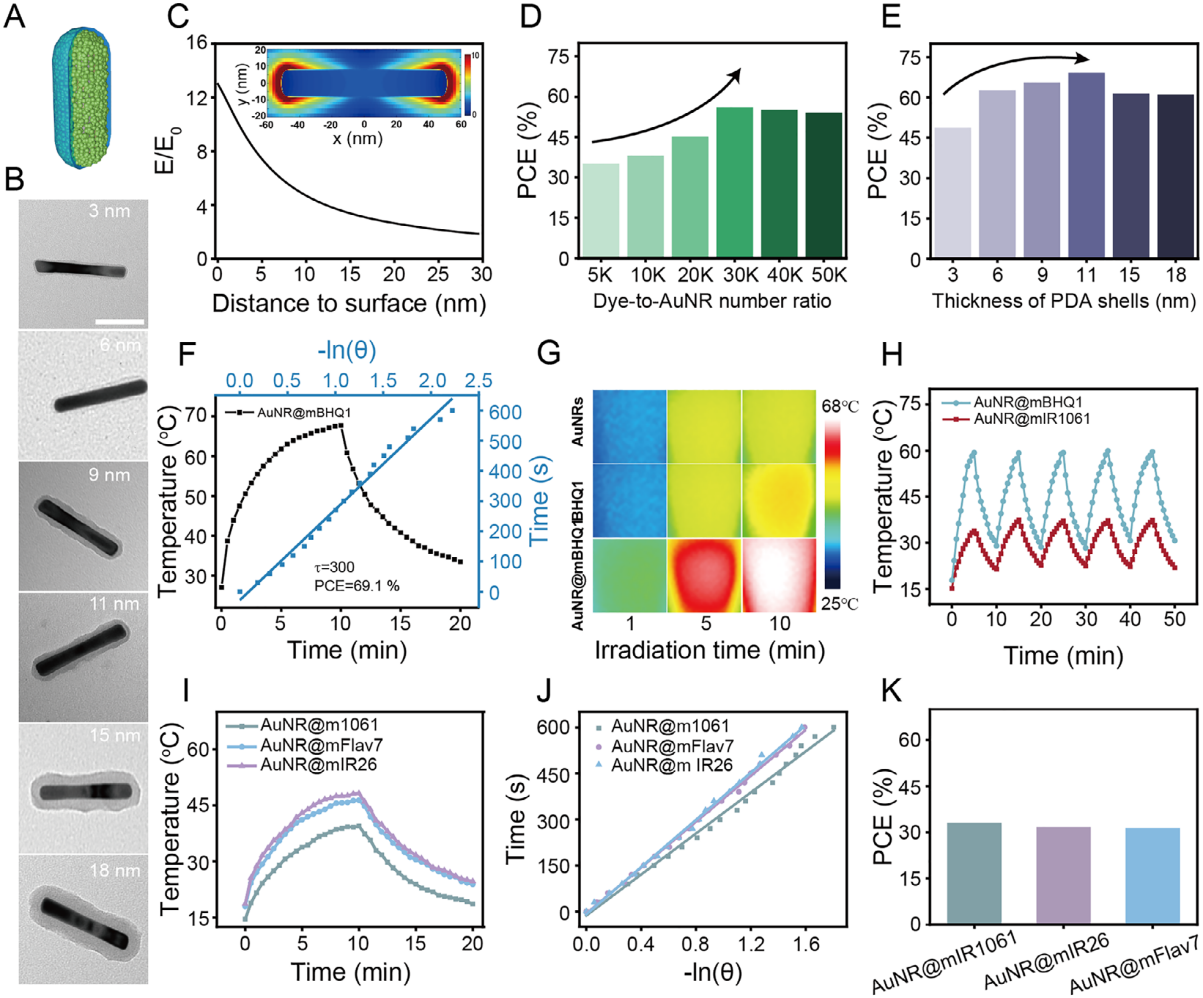
(A) Cartoon of AuNRs loading multilayer BHQ1 dyes within a PDA shell. (B) TEM images of AuNR@mBHQ1 NPs with thickness of, acquired from the dopamine concentrations of 0.05, 0.10, 0.15, 0.20, 0.25, and 0.30 mg/mL.Scale bar = 50 nm. (C) FDTD stimulation of the relationship of field enhancement with the distance from the surface of AuNRs and the field distribution of single AuNRs at an excitation wavelength of 1064 nm (the inset). (D) PCE optimization of AuNR@mBHQ1 by varying the dye‐to‐AuNR number ratio. The PDA shell thickness in this case is fixed at ca. 6 nm. (E) PCE optimization of AuNR@mBHQ1 NPs by varying PDA shell thickness. The dye‐to‐AuNR number ratio is sufficient. (F) Heating–cooling curve of the aqueous suspension of 200 µg/mL probes and the corresponding plot of t vs. ‐ln(θ) during the cooling period (1064 nm laser, 1.0 W/cm^2^). (G) Infrared thermal images of AuNRs and AuNR@mBHQ1 NPs after laser irradiation (1064 nm, 1.0 W/cm^2^) for 10 min. (H) Photothermal stability of the conjugates after five heating–cooling cycles. (I) Heating–cooling curves, (J) cooling functions curve, and (K) corresponding PCE of AuNR@m1061, AuNR@mFlav7, and AuNR@mIR26 NPs. The average number of confined reporter molecules per AuNR in three NPs in panel K is ca. 17426.

Encouraged, we further harnessed the local fields to improve the PCE of the BHQ1‐plasmon hybrid NPs with multilayer dyes, which we referred to as AuNR@mBHQ1 NPs (Figure 4A). Beyond SAMs of dyes, we encapsulated more dyes into the hotspot regions around plasmonic nanostructures with the assistance of polydopamine (PDA) [48], a highly adhesive and stable polymer coating. To this end, BHQ1, dopamine, and AuNRs were mixed for a one‐pot reaction to obtain dye‐incorporated PDA‐coating NPs. After encapsulation of BHQ1 within PDA onto AuNRs, transmission electron microscopy (TEM) images clearly demonstrated uniform PDA coating on AuNRs, with shell thicknesses varying from 3 to 18 nm (Figure 4B; Figure S4). Accordingly, the UV–vis–NIR spectra of the prepared PDA‐coated NPs exhibit a gradual red shift (Figure S5).

Since the electromagnetic fields of AuNRs exist on their surface at tens of nanometers exceeding 20 nm, as illustrated by the field distribution of a single AuNR with the finite‐difference time‐domain (FDTD) stimulation method (Figure 4C), such a PDA coating within dyes will take full advantage of the field area to magnify non‐radiative transition of BHQs beyond SAMs length (ca. 2 nm). With this method, we mainly manipulated both parameters to maximize the PCE of AuNR@mBHQ1 NPs. First, we fixed the thickness of PDA (ca. 6 nm) to tune the BHQ1‐to‐AuNRs ratios. When the ratio of BHQ1 to AuNRs augments from 5 to 30k, the PCE gradually improves from 35% to 56% (Figure 4D). However, as the ratio of BHQ1 to AuNRs continues to increase to 40k, the PCE remains slightly declining, maybe because the excessive dyes exceed the encapsulation capacity of the PDA shell [49]. Subsequently, we tuned the PDA thickness in the presence of sufficient BHQ1 dyes (BHQ1‐to‐AuNR ratio of > 30k). The calculated amounts of three BHQs inside plasmonic nanostructures are shown in Table S2. As shown in Figure 4E, the increase in PDA thickness from 3 to 11 nm results in a marked improvement in the PCE of the hybrids from 48.5% to 69.1%. Notably, the maximal PCE (69.1%) was achieved with a PDA thickness of 11 nm. As the PDA thickness increases up to > 15 nm, the PCE declines, which may be because thick PDA destabilizes the NPs and thus leads to precipitation, as evidenced by the TEM images and absorption (Figures S4 and S5). Thus, we adopted both optimal conditions in the following experiments, e.g., the PDA thickness of ca. 11 nm and BHQ1‐to‐AuNR ratio of 30k. Through analysis of one cyclic temperature profile, we calculated the PCE as high as 69.1% (Figure 4F). The PDA shell contributes negligibly to the overall photothermal performance of the probes (data not shown), owing to the negligible absorption in the NIR‐II region [50]. Furthermore, as the NPs concentration increases, the temperature of the hybrid NPs shows a robust elevation (Figure S6). Infrared thermal images intuitively visualized the superior photothermal conversion capacity of AuNR@mBHQ1 NPs, significantly outperforming the other two species (Figure 4G). In short, the plasmonic field of AuNRs effectively modulates the photothermal efficiency of BHQ‐decorated hybrid NPs and thus achieves BHQ‐enhanced plasmonic photothermal conversion. In addition, five irradiation cycles failed to cause any noticeable change in either temperature or nanosheet morphology (Figure 4H), attesting to the outstanding stability of the resulting AuNR@mBHQ1 NP hybrids.

To demonstrate the versatility of this method, we also tested the PCE of AuNSs and AuNPs with multilayered BHQ2 and BHQ3 molecules, respectively. We prepared two other types of nanoprobes, i.e., AuNS@mBHQ2 and AuNP@mBHQ3 NPs, using the aforementioned method (Figure S7). TEM shows that the PDA shell thickness ranges from approximately 2–12 nm for AuNS@mBHQ2 NPs and 2–12 nm for AuNP@mBHQ3 NPs. Meanwhile, the UV absorption of the PDA‐coated NPs also gradually redshifted (Figure S8). First, we fixed the PDA thickness at ca. 4 nm and found that the highest PCE was achieved when the ratio of BHQ2 to AuNSs was 50k (Figure S9A). Of course, an excessive BHQ2‐to‐AuNS ratio of ≥ 50k would lead to a decrease in PCE, for the reason mentioned above. Second, with sufficient BHQ2 molecules present, we achieved the highest PCE of the NPs to 63.9% by adjusting the thickness of the PDA shell through varying dopamine concentrations (Figure S9B). Likewise, manipulating the PCE of AuNP@mBHQ3 NPs by varying the dye‐to‐AuNP ratio and PDA thickness achieves the optimal PCE as high as 43.9% under visible laser irradiation (Figure S9C,D), which is higher than that of AuNP@sBHQ3 NPs. Overall, both PCE optimization experiments for AuNS@mBHQ2 and AuNP@mBHQ3 NPs well support the universality of this strategy to boost the whole PCE of plasmonic nanostructures by add‐on BHQs.

On the other hand, we also ruled out the influence of PDA on whole photothermal efficacy, which shows negligible absorption in NIR‐II and small absorption in NIR‐I and the visible region [50]. In the absence of BHQ molecules, the PCE of AuNR@PDA, AuNS@PDA, and AuNP@PDA NPs (31.2%, 37.4%, and 16.3%, respectively) are close to or slightly higher than that of pure AuNRs, AuNSs, and AuNPs (27.5%, 27.4%, and 18.7%, respectively, Figure S10), indicating that the negligible effect on the total photothermal efficacy of hybrid plasmonic nanostructures. Since the PCE displays a nonlinear response to electric field, we proposed this nonlinear photothermal effect as a new concept derived from the synergistic amplification of BHQ molecules and plasmon as a result of field‐molecule coupling (rather than a simple intensity‐dependent effect), which may guide us to develop new plasmonic photothermal reagents.

It was noteworthy that encapsulation of the three usual fluorescent dyes (IR1061, IR26, Flav7) inside PDA onto the surface of AuNRs shows a slightly higher PCE (32.9%, 31.6%, and 31.3%, respectively) than the AuNRs (27.5%, Figure 4I–K), implying that this synergistic effect is not applicable for fluorescent resonant molecules. Similarly, calculating the PCE of these fluorescent dye‐based NPs in the NIR‐I and visible region proves that the PCEs of these NPs are only slightly higher than those of normal AuNSs or AuNPs (Figure S11). Taken together, the effectiveness of this strategy does not rely on the shape or makeup of plasmonic materials, yet it markedly boosts their photothermal performance through the rational combination of BHQs with plasmonic fields.

### 2.5 In Vitro Photothermal Ablation of Cancer Cells

These molecule‐plasmon hybrids, AuNR@mBHQ1 NPs, thus served as photothermal reagents for PPT in the NIR‐II window. Over a period of 7 days, the hydrodynamic size tests indicate that AuNR@mBHQ1 NPs can stably exist under PDA coatings with thicknesses ranging from 3 to 11 nm (Figure S12). Therefore, AuNR@mBHQ1 nanoprobes with optimized conditions (PCE of 69.1%), i.e., PDA thickness of 11 nm and BHQ1‐to‐AuNR ratio of 30k, were adopted for follow‐up experiments. To this end, cellular viability of both 4T1 and MCF‐7 cells incubated with the hybrid NPs of various concentrations for 48 h was first examined. It is found that over 89% of both cells survive even at a concentration of 2 nM (Figure S13), indicating negligible cytotoxicity and excellent biocompatibility.

**FIGURE 5:**
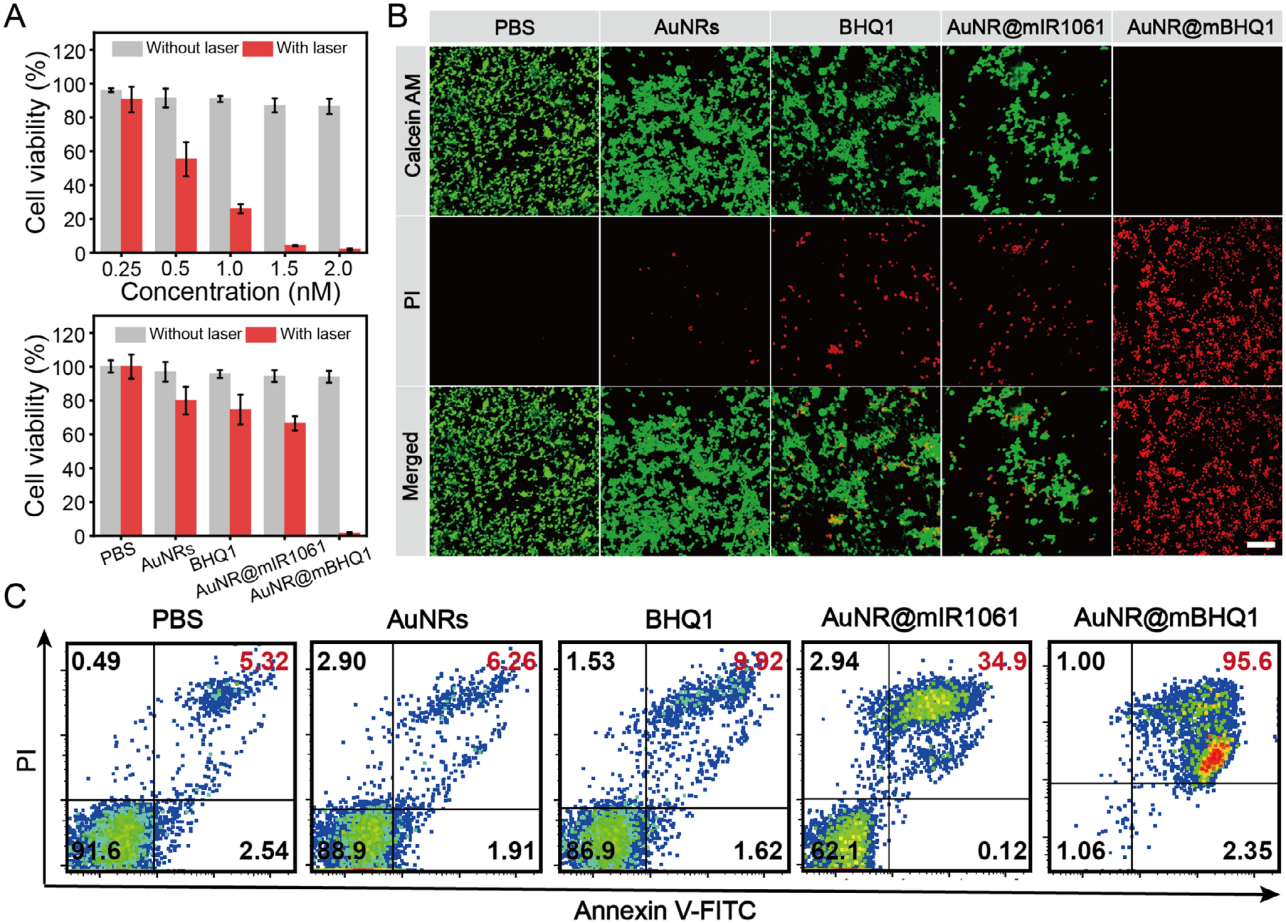
(A) Cell viability of 4T1 cells incubated with (up row) AuNR@mBHQ1s NPs at various concentrations and (bottom row) five group treatments at corresponding concentrations in the dark and after NIR‐II laser irradiation (1064 nm, 1.0 W/cm^2^, 5 min). (B) Calcein AM (green) and propidium iodide (red) co‐staining fluorescence imaging of 4T1 cells receiving different treatments. Scale bar = 200 µm. Green fluorescence represents live cells, and red indicates dead ones. (C) Flow cytometry analysis of apoptosis and necrosis of 4T1 cells receiving five different treatments.

The efficacy of photothermal ablation was determined in vitro by incubating 4T1 cells with these AuNR@mBHQ1 NPs of various concentrations, followed by irradiating with a 1064 nm laser (1.0 W/cm^2^). As expected, with the increase of NP concentration, more cells were killed after the laser irradiation (Figure 5A, upper row). At a concentration of 2.0 nM, the cell viability decreased to 2%. Moreover, compared to the other four examined groups (PBS, AuNRs, BHQ1, AuNR@IR1061 NPs), the AuNR@mBHQ1 NPs demonstrated the highest cell killing efficiency under the same conditions. After 5 min of irradiation, the viability of 4T1 cells incubated with AuNRs and AuNR@IR1061 NPs is reduced to 79% and 66%, while only 1.5% of cells treated with AuNR@mBHQ1 are viable (Figure 5A, bottom row). These results display the remarkable photothermal effect of the hybrid plasmonic NPs with add‐on BHQ molecules that accelerates cell killing.

Live–dead cell staining with calcein AM (green) and propidium iodide (PI, red) confirmed that cell death occurs only in laser‐illuminated areas, visualizing the phototherapy efficacy of AuNR@mBHQ1 NPs. Confocal fluorescent imaging of cells shows that green fluorescence was observed in the PBS, AuNRs, BHQ1, and AuNR@IR1061 NPs groups. On the contrary, the red fluorescence (Figure 5B) reveals that the hybrid NPs lead to total destruction of 4T1 cells upon NIR laser irradiation (1.0 W cm^−2^, 5 min), clearly showing strong photocytotoxicity. These outcomes confirm the high efficacy of photothermal ablation of cancer cells through hyperthermia induced by AuNR@mBHQ1 NPs. Flow cytometry was used to analyze apoptosis and necrosis in 4T1 cells subjected to different treatments. As shown in Figure 5C, the cell survival rate for AuNR@mBHQ1 was 1.06%, which was lower than that of AuNRs (88.9%), BHQ1 (86.9%), and AuNR@mIR1061 (62.1%), suggesting the best cell killing ability. Thus, the cell death is ascribed to apoptosis and necrosis [51].

### 2.6 In Vivo Photothermal Therapy and Biosafety Evaluation

To highlight the superiority of deep‐tissue PPT in NIR‐II windows, we studied the photothermal penetration depth of AuNR@mBHQ1s NPs in NIR‐I and NIR‐II windows using chicken breast muscle as the model. The experimental setup is shown in Figure S14A. The penetration depth through the muscle thickness is 10 mm in the NIR‐II region at 1064 nm, greater than that in the NIR‐II region at 808 nm (8 mm, Figure S14B). Furthermore, compared with the temperature change induced by the 808 nm laser, the temperature decay trend for the 1064 nm laser over tissue thickness is slower, suggesting a superior photothermal heating capacity of AuNR@mBHQ1 NPs in deep tissue in the NIR‐II.

**FIGURE 6:**
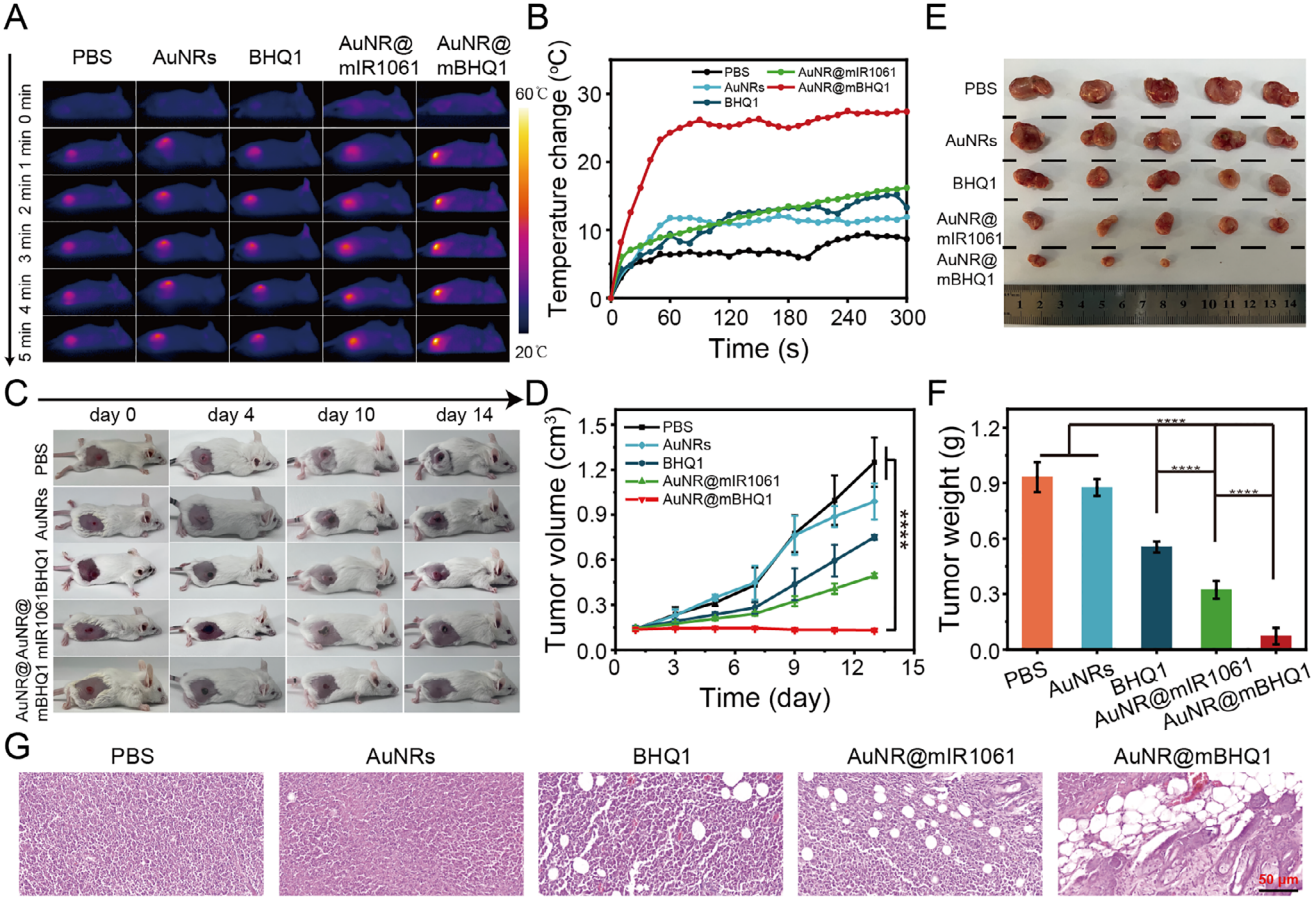
(A) Thermal images and heating temperatures (at the tumor site) of tumor‐bearing mice during continuous laser radiation (1064 nm, 1.0 W/cm^−2^) for 5 min after post‐injection of AuNR@mBHQ1 NPs, and (B) corresponding temperature increase curve at the tumor site of mice. (C) Tumor changes of the mice after photothermal therapy in these five groups. Mice were irradiated with a 1064 nm laser at a power of 1 W /cm^−2^. (D) Relative tumor volume of the mice during the five treatments. (E) Photograph of the excited tumor tissue (*n* = 5 for each group) after the different treatments, and (F) corresponding tumor weights. (G) H&E staining of the tumor tissues treated with these five groups. Values were expressed as mean ± SD, and the statistical analysis was determined by ANOVA, ^****^ *p* < 0.0001.

Inspired by the above results, photothermal ablation of cancer cells was further investigated in vivo. Upon intravenous administration of the NPs, an infrared thermal camera recorded real‐time temperature changes at the tumor site (Figure 6A). Within 3 min of 1064 nm laser exposure, the tumor temperature in the NP treated group sharply rose from 32°C to 59.4°C, sufficient for malignant tumor ablation (Figure 6B), while AuNRs‐treated mice showed only a modest rise in temperature by ca. 13.3°C. Next, the tumor sizes after five treatments plus laser irradiation were continuously monitored for 16 days, and the visual photographs of tumor tissues were shown in Figure 6C. After 14 consecutive days of precise monitoring and detailed observation of tumor size, the other three groups showed fast tumor growth, demonstrating that laser irradiation minimally influenced tumor growth rate. By comparison, the tumor volume fluctuation in the AuNR@mBHQ1 group strictly maintains to an extremely small range of 0.03 mm^3^ (Figure 6D), confirming that AuNR@mBHQ1 NPs are highly effective in inhibiting tumor growth, thanks to the BHQ‐augmented photothermal hybrid NPs.

After the end of the 14‐day PTT period, we meticulously followed the predetermined protocol to carefully remove the tumor tissues from the experimental mice for direct comparison. It is worth noting that we chose a sample size of *n* = 5 per group, which fully complies with the 3Rs principle (Reduce, Refine, and Replace) [52, 53]. This step not only allows us to visually observe that the tumors in the AuNR@mBHQ1 group exhibit the smallest volume compared to other control groups (Figure 6E). We further weighed these tumor tissues; the tumors in the AuNR@BHQ1 group are also the lightest in weight (Figure 6F), further reaffirming the above observations. Additionally, histological analyses with hematoxylin and eosin (H&E) were implemented, which reveals the AuNR@BHQ1 treated tissue exhibited the most cell nucleus damage (Figure 6G). These outcomes further underscore the remarkable photothermal efficacy of AuNR@mBHQ1 in inhibiting tumor growth under a 1064 nm laser. Over the entire treatment course, NP administration and related interventions barely affected the body weight of the mice (Figure S15), which points to favorable biosafety during treatment.

We ultimately investigated the potential side effects of AuNR@mBHQ1 hybrid NPs in vivo by intravenously injecting them (200 µL, 1.0 nM) into healthy mice. First, we analyzed blood biochemistry parameters of mice after 15 days, focusing on the indicators including alanine aminotransferase, aspartate aminotransferase, and blood urea nitrogen levels, which indicate that the administration of the NPs has no significant impact on liver and kidney function compared to that in PBS‐treated mice (Figure S16A). Additionally, by examining the key hematological indicators of the treated mice, including red blood cell count, hemoglobin content, and so on, it is found that the examined parameters are similar to those in the normal status in PBS‐treated mice (Figure S16B). Furthermore, we conducted histological examinations with standard H&E staining on the main organs isolated from the treated mice, such as the heart, liver, spleen, lungs, and kidney. No inflammatory and pathological abnormalities were found in both NPs and PBS‐treated groups (Figure S17). Overall, the above results exemplify the satisfactory biocompatibility and biosafety of the hybrid NPs, which could serve as a promising nanoplatform for cancer photothrombotic.

To quantitatively evaluate the biodistribution of AuNR@mBHQ1 NPs in mice, the Au content in tumors and major organs (liver, spleen, kidney, heart, lung) was measured at 6 h, 12 h, 7 d, and 28 d post‐injection using inductively coupled plasma mass spectrometry (ICP‐MS) in Figure S18. The time‐dependent profiles revealed a peak tumor accumulation of ca. 4% ID/g at 12 h, markedly higher than the levels at 6 h (ca. 1.5% ID/g) and 7 d (ca. 1% ID/g), confirming effective tumor targeting and subsequent clearance. Meanwhile, elevated Au levels in the liver, spleen, and kidney compared to the heart and lung indicated intrinsic uptake by the mononuclear phagocyte system (MPS). We further investigated the pharmacokinetics and clearance of AuNR@mBHQ1 NPs. The blood circulation curve (Figure S19A) shows that 88% of AuNR@mBHQ1 NPs are cleared from the blood within 12 h, supporting our choice of laser irradiation at 12 h post‐administration—when tumor enrichment peaks (ca. 4% ID/g). Moreover, approximately 28% of the probes are excreted within 28 days (Figure S19B), indicating fecal elimination [54]. The residual Au content at the tumor site significantly decreased to below 1% ID/g by 28 days, confirming effective clearance of AuNR@mBHQ1 NPs from tumor tissues without long‐term accumulation. These outcomes reveal that PDA‐coated hybrid NPs persist in the liver and spleen for a relatively long period because the PDA shells remain stable in vivo for one month [55]. Such high stability benefits the efficacy of carried drugs/NPs [56]. Although there is a balance between EPR‐mediated targeting and sequestration within the MPS, the biological distribution and blood profiles data indicate that our design has no significant impact on biosafety. Of course, biomimetic strategies such as cell membrane encapsulation can further optimize their tumor uptake efficiency and improve long‐term biosafety of AuNR@mBHQ1 NPs [57, 58].

## 3 Conclusions

In summary, we report a universal method to dramatically improve plasmonic photothermal conversion, which we termed BHQ‐enhanced plasmonic photothermal conversion. The PCE of the resultant hybrids is much higher than that of normal plasmonic NPs, and it can be further increased through adding the number of BHQ molecules to electromagnetic hotspots, leading to a PCE as high as 69.1% for AuNR@mBHQ1 NPs. Such an enhanced photothermal conversion can be attributed to the dual‐component synergy of plasmon and BHQ molecules. This method is not only suitable for plasmonic nanostructures regardless of the size, morphology, and composition, but also can be integrated with current strategies such as plasmonic blackbody [59], broadband plasmon [60], Tamm plasmon [61], dark plasmon [62], to further advance the photothermal performance for extensive applications. This phenomenon cannot be realized by fluorescent molecules, which usually result in radiative transitions enhancement rather than non‐radiative heat generation. Furthermore, both in vitro and in vivo experiments have confirmed that these BHQ‐modified plasmonic NPs are superior photothermal agents to their unmodified counterparts, allowing for efficient hyperthermia ablation of deep‐seated tumors in the NIR‐II window. This work not only guides the design of NIR‐II photothermal reagents by rationally manipulating electromagnetic fields with BHQs to perform wavelength‐specific phototheranostics at high efficiency, but also inspires the exploitation of new phenomena from BHQ‐plasmon interactions at the single‐molecule level [63, 64].

## 4 Material and Methods

### 4.1 Materials and Instruments

All chemicals were purchased from commercial suppliers and used without further purification. Cy641 was synthesized based on our previous work [31]. Hydroxylamine hydrochloride, sodium citrate, and sodium hydroxide, crystal violet, rhodamine B, Rh6G, and IR1061 were obtained from Sigma–Aldrich. IR26, Flva7, IR783, ICG, and Cy641 were obtained from Xi'an Ruixi Biotechnology Co., Ltd. Dopamine hydrochloride, trisodium citrate, and L‐ascorbic acid (98%) were obtained from Aladin Co., Ltd. (Shanghai, China). Hydrogen tetrachloroauratehydrate (HAuCl_4_·4H_2_O) was obtained from Sinopharm Chemical Reagent Co., Ltd. eBioscience flow cytometry staining (FACS) buffer was bought from Thermo Fisher Scientific. The 808 and 1064 nm laser devices with a power density of 1.0 W/cm^2^ were obtained from Changchun New Industries Optoelectronics Technology Co., Ltd. The 633 nm laser device with a power density of 1.2 W/cm^2^ was obtained from Dongguan Fulei Laser Technology Co., Ltd. TEM images were acquired on a JEM‐1400 instrument (JEOL, DBPR) at an accelerating voltage of 3.0 kV. A Lambda 950 UV–vis–NIR spectrophotometer (Shanghai, China) was used to acquire steady‐state absorption spectra. Transient absorption spectra were performed using a HARPIA‐TA instrument (Light Conversion), and sample solutions (ca. 1.0 mM) were measured in a 1 mm quartz cuvette at room temperature.

All procedures were carried out using distilled water (18.0 MΩ·cm).

### 4.2 Absolutely Fluorescence Quantum Yield (QY) Test

For AQY measurements, an integrating sphere (Thorlabs; IS200) distributed the incoming light across its entire inner surface via multiple reflections [32, 65]. A quartz cuvette at the integrating sphere's center held the colloidal samples, which were then excited by lasers of specific wavelengths (1064, 785, and 633 nm for BHQ1, BHQ2, and BHQ3, respectively). The QY is defined as:

(1) $$\mathrm{QY}=\frac{photonsemitted}{photonsabsorbed}=\frac{E\left[\mathrm{sample}\right]}{L\left[\mathrm{blank}\right]-L\left[\mathrm{sample}\right]}$$

where QY, *E[sample]*, *L[blank]*, and *L[sample]* represent the quantum yield, emission intensity, and excitation light intensities for water and BHQs, respectively.

### 4.3 Preparation and Optimization of Three Kinds of Plasmonic Nanostructures

NIR‐II AuNRs (106 × 16 nm), NIR‐I AuNSs (∼60 nm) and visible AuNPs (60 nm in diameter), with plasmon resonance peaks of ca. 1050, 693, and 552 nm were prepared following the references [66, 67]. For optimization, CTAB‐capped AuNRs were first centrifuged to lower the CTAB concentration to 10 mM. Next, mPEG‐SH (1 µM, MW ∼1 kDa) was added to replace positively charged CTAB on the surface of AuNR with neutral mPEG‐SH. To minimize colloid aggregation, the molar ratio of mPEG‐SH to AuNR1050 was adjusted to ca. 10% of the theoretical maximum coverage area, as described in reference [49]. After repeated dilution and centrifugation, the total CTAB in the solution was greatly reduced until the zeta potential approached zero.

### 4.4 Preparation of BHQ‐plasmon Hybrids With SAMs of Dyes

For AuNR@sBHQ1 NPs, the AuNRs were treated with dropwise addition of the DMF dye solution in a vast excess (> 50 000:1) for adequate adsorption. Following several centrifugation cycles, the resulting conjugates were stored for further use. For AuNS@sBHQ2 NPs, AuNSs (1 nM, 1 mL) were first conjugated to mercaptoethylamine (1 mg/mL, 30 µL) via an Au─S bond, followed by reacting with BHQ2 (0.1 mM, 20 µL) between NHS and NH_2_. For AuNP@sBHQ3 NPs, AuNPs (1 nM, 1 mL) were first conjugated to mercaptoacetic acid via an Au─S bond, followed by reacting with BHQ3 (0.1 mM, 20 µL) between COOH and NH_2_ in the presence of EDC (25 mM, 5 µL) through EDC/NHS chemistry.

### 4.5 Preparation of BHQ‐Plasmon Hybrids With Multilayer Dyes

The optimization method for three BHQ molecules was similar, and that for BHQ1 was described. First, with fixed dopamine concentration (0.14 mg/mL), we mixed BHQ1 molecular solution with AuNRs at the pre‐determined dye‐to‐AuNR ratio ranging from 20 to 120k. Afterward, the mixture was thoroughly stirred for 5 h to obtain the probes with multilayer dyes. Second, in the case of excess dye (dye‐to‐AuNR ratio greater than 30 k), dopamine solutions of different concentrations of 0.05, 0.1, 0.15, 0.20, 0.25, and 0.30 mg/mL were added to AuNRs. After multiple centrifugations, the resultant AuNR@mBHQ1 NPs with various thickness shells were obtained.

### 4.6 Calculation of Dye Amounts Around Plasmonic Nanostructures

In brief, the molecules (BHQ1, BHQ2, and BHQ3) with a given concentration were added to the absorption‐matched nanostructured solutions with intense stirring. After 12 h incubation, the mixed solution was centrifuged to obtain the supernatant, followed by quantification through the absorbance peaks (982 nm for BHQ1, 785 nm for BHQ2, and 564 nm for BHQ3, respectively) according to the previous well‐established standard curves in our references [18, 26, 31]. For the SAMs method, the theoretical maximum coverage amounts in SAM‐based tags were calculated corresponding to a footprint area of 2.0 nm^2^ for each BHQ molecule. Calculation results of the three BHQs amounts inside plasmonic nanostructures were shown in Table S1.

### 4.7 PCE (η)

The PCE was calculated following the method reported in the literature [68, 69]. Detailed details are described in the Supporting Information.

### 4.8 Computational Details

All DFT calculations were carried out by the Vienna Ab initio Simulation Package [14]. The exchange‐correlation potential was described by the Perdew–Burke–Ernzerhof generalized gradient approximation, and electron‐ion interactions were treated with the projector augmented wave (PAW) method. A cutoff energy of 400 eV was used, and Brillouin zone integration employed a 3 × 3 × 1 Monkhorst‐Pack k‐point grid. The self‐consistent iteration convergence criteria were set to 10^−4^ eV for energy and −0.02 eV·Å^−1^ for force.

To accurately depict the magnetic properties of the studied systems, spin polarization was explicitly incorporated into all calculations. The magnetic moments were determined by integrating the spin density within the PAW spheres. For systems containing nickel (Ni)—a metal that can exhibit substantial magnetization—the initial magnetic moments were set to their high‐spin configurations. To analyze the electronic properties of the systems, the density of states (DOS) was calculated [41]. The projected density of states was decomposed into contributions from specific atoms (including H, C, S, Ni, and Au) and their respective orbitals. This decomposition allowed for an in‐depth investigation of the roles these atoms and orbitals play in adsorption processes and surface interactions. For DOS calculations, a finer k‐point grid with a 5 × 5 × 1 setup was used to guarantee high resolution of the results.

The charge transfer occurring during adsorption was analyzed by computing the charge density difference (Δρ), which is defined as follows:

(2) $$\Delta \rho =\rho^{*}\;\left(A+\mathrm{sub}\right)-\rho \left(\mathrm{sub}\right)-\rho \left(A\right)$$

Here, ρ^*^(A+sub) represents the total charge density of the adsorption system, ρ(sub) denotes the charge density of the clean substrate, and ρ(A) stands for the charge density of the free adsorbate. The isosurfaces of Δρ were visualized to identify regions where charge accumulation and depletion take place.

The adsorption energy (*E*_ads_) of adsorbate BHQ was defined as:

(3) $$E_{\mathrm{ads}}=E_{*\mathrm{BHQ}}-E_{\mathrm{BHQ}}-E_{\mathrm{sub}}$$

where E_*BHQ_ represents the energy of BHQ molecules adsorbed on the surface. *E*_sub_ is the energy of a clean surface; *E*_BHQ_ represents the energies of BHQ.

After completing the geometric optimization of the adsorption configuration of BHQ onto the Au(111) surface, the differential charge density was obtained by subtracting the respective electronic charge densities of BHQ and Au(111) from the electronic charge density of the total adsorption system [38]. The isosurface value was set to 0.003 e/Bohr^3^.

### 4.9 Cell Culture and Cytotoxicity Study

Mouse breast cancer 4T1 cells (Wuhan Pricella Biotechnology) were cultured in 1640 medium with 10% fetal bovine serum and 1% penicillin–streptomycin at 37°C in 5% CO_2_ under humidified conditions. For dark cytotoxicity assessment, cells were seeded into 96‐well plates (5 × 10^3^ cells/well) and incubated for 24 h. Next, the cells were incubated with PBS, AuNRs, BHQ1, AuNR@IR1061, and AuNR@mBHQ1 NPs in the dark, respectively. Following a further 12 h incubation, the medium was aspirated, followed by three PBS washes of the cells. Then, the 4T1 cells were incubated in the dark with a fresh serum‐free medium containing CCK‐8 (10%) for 2 h. Finally, the absorbance of the resulting product at 450 nm was recorded by a microplate reader. The relative cell viability was determined according to the following Equation (4):

(4) $$\mathrm{Cell}\;\;\mathrm{viability}\;\;\left(\%\right)=\frac{OD_{\mathrm{ps}}-OD_{\mathrm{blank}}}{OD_{\mathrm{control}}-OD_{\mathrm{blank}}}\times 100\%$$

### 4.10 In Vitro Photothermal Ablation of Cells and Live/Dead Staining

First, 4T1 cells were plated into six confocal dishes and left to incubate for 24 h. Then, the cells were subjected to different treatments: PBS, AuNRs (50 µg/mL), BHQ3 (10 µM), AuNR@mIR1061 (50 µg/mL) and AuNR@mBHQ1 NPs (50 µg/mL). After 12 h of incubation, the cells were exposed to a 1064 nm NIR laser (0.5 W/cm^2^) for 5 min. Following different treatments, the cells were incubated at 37°C for 30 min and then gently washed twice with PBS buffer. Subsequently, the cells were stained with PBS containing calcein‐AM and PI for 30 min. The incubation solution was then discarded, and the cells were gently washed twice with PBS to remove any residual dyes. All samples were then imaged using a confocal microscope.

### 4.11 Cell Apoptosis Analysis by Flow Cytometry

First, a density of 2 × 10^5^ 4T1 cells per well was seeded into 6‐well plates, followed by 24 h of culture. The cells were then subjected to different treatments: PBS, AuNRs (2 nM), BHQ1 (10 µM), AuNR@mIR1061 (2 nM) and AuNR@mBHQ1 NPs (2 nM). The following procedure was performed after a 12 h incubation: cells were collected, exposed to a 1064 nm laser (0.5 W/cm^2^) for 5 min, and then held at 4°C for an additional 30 min. Subsequently, the cells underwent a PBS wash followed by centrifugation at 1000 rpm for 5 min at 4°C. The resulting pellet was stained using an Annexin V‐FITC/PI apoptosis kit as per the manufacturer's instructions, and the stained cells were subjected to FACS analysis.

### 4.12 Tumor Model

The Ethics Committee for Animal Experiments of Yantai Institute of Coastal Zone Research, Chinese Academy of Sciences, approved all procedures (No. KJ‐LL‐011). Female BALB/c mice, aged 6–8 weeks and weighing 18–22 g, were supplied by Hangzhou Ziyuan Experimental Ltd. (Hangzhou, China) and maintained under pathogen‐free conditions with standard chow and water. To generate tumor‐bearing models, each mouse received a subcutaneous injection of 4T1 cells (1 × 10^6^ cells) suspended in PBS. When tumors reached a volume of 100–150 mm^3^, the mice were randomly divided into groups for further experiments.

### 4.13 In Vivo PTT of Tumors

Photothermal therapy was performed on tumor‐bearing mice randomly assigned to five groups (n = 5). Each group was intravenously given one of the following: PBS control, AuNRs (1 nM), BHQ1 (100 µM), AuNR@mIR1061 NPs (1 nM), or AuNR@mBHQ1 NPs (1 nM). Approximately 12 h later, the mice received 5 min of 1064 nm laser exposure, and an NIR thermal camera (1.0 W/cm^2^) recorded in vivo photothermal images. Bilateral measurements of tumor size and body weight were taken every two days. After 14 days, the mice were sacrificed; their excised tumors underwent H&E staining to quantify cell death.

### 4.14 ICP‐MS Analysis‐

Organs and tumors were collected at 6 h, 12 h, 7 d, and 28 d after intravenous injection of AuNR@mBHQ1 NPs, digested in aqua regia, and analyzed for Au content using an inductively coupled plasma mass spectrometer. The percentage of injected dose per gram of tissue (%ID/g) was calculated for each organ.

## Author Contributions

J.L. and R.Z. designed this study, performed a majority of experiments, and prepared the manuscript. X.C. and L.S. prepared the AuNPs, AuNSs, and AuNRs.

## Conflicts of Interest

The authors declare no conflicts of interest.

## Supporting information




**Supporting File**: advs76680‐sup‐0001‐SuppMat.docx.

## Data Availability

The data that support the findings of this study are available from the corresponding author upon reasonable request.
