# Supplementary material for: Black Hole Quencher‐Enhanced Plasmonic Photothermal Conversion
Source: Adv Sci (Weinh). 2026 Jul 14;13(57):e76680. doi: 10.1002/advs.76680 (PMC13367111; doi:10.1002/advs.76680)
Supplement: Supplementary file 1 [file ADVS-13-e76680-s001.docx]

**Supporting Information**

**Black Hole Quencher-Enhanced Plasmonic Photothermal Conversion**

Ruiyuan Zhang^1,3^, Xinru Chen^2,3^, Lin Shen^3^ and Jin Li^2,3*^

^1^School of Information and Electronic Engineering, Shandong Technology and Business University, Yantai 264005, China

^2^Faculty of Biomedical Engineering, Shenzhen University of Advanced Technology, Shenzhen 518107, China

^3^CAS Key Laboratory of Coastal Environmental Processes and Ecological Remediation, Yantai Institute of Coastal Zone Research, Chinese Academy of Sciences, Yantai 264003, China

**Corresponding author:* lijin@suat-sz.edu.cn

Table of contents

1. Supplementary experimental section
2. Supplementary Figures S1-S19

**Supplementary experimental section**

**Photothermal conversion efficiency (PCE, η)**

Typically, 1.0 mL of all test solutions (water, BHQ1, AuNR, AuNR+BHQ1, AuNR@BHQ1, and AuNR@mIR1061 NPs) were continuously irradiated with a 1064 nm laser (1.0 W/cm²) until the temperature reached a steady state, followed by natural cooling to ambient temperature. The temperature was measured every 30 s using an infrared thermal imaging camera (TESTO-865). Water was used as a control. The PCE (η) was calculated using the following equation (1):

$$\begin{aligned} \text{η}\text{=}\frac{\text{hs}\left( \text{T}_{\text{max}}\text{-}\text{T}_{\text{surr}} \right)\text{-}\text{Q}_{\text{dis}}}{\text{I}\left( \text{1-}\text{10}^{{\text{-}\text{A}}_{\text{808 nm}}} \right)}\#\text{(}\text{1}\text{)} \end{aligned}$$

where h is the heat transfer coefficient, S is the surface area of the container, *T*_max_ and *T*_surr_ are the maximum temperature reached and the ambient temperature, respectively, and *Q*_dis_ represents the heat dissipated by the solvent and container due to laser irradiation. The value of *hs* is calculated from the following equation (2):

$$\begin{aligned} \text{hs}\text{=}\frac{\text{mC}}{\text{τ}}\#\left（ \text{2} \right） \end{aligned}$$

where m and C represent the mass and heat capacity of water (C_water_ = 4.2 J·g^-1^·℃^-1^), respectively, and τ is the time constant for heat transfer, which is determined by the equation (3):

$$\begin{aligned} \text{t}\text{=-τ}\ln\left( \text{θ} \right)\#\text{(}\text{3}\text{)} \end{aligned}$$

where t represents time, and θ is the dimensionless driving force for the temperature decline cycle. The value of θ is obtained from the below equation (4):

$$\begin{aligned} \text{θ}\text{=}\frac{\text{T}_{\text{RT}}\text{-}\text{T}_{\text{surr}}}{\text{T}_{\text{max}}\text{-}\text{T}_{\text{surre}}}\#\text{(}\text{4}\text{)} \end{aligned}$$

where *T*_RT_ represents the real-time temperature during the cooling period.

**Biosafety assays**

Healthy female BALB/c mice that were divided into two groups (n = 5 each group). The first group was intravenously injected with PBS (100 μL, pH = 7.4) as control, while the second group was intravenously injected with AuNR@mBHQ1 NPs (100 μL). All mice were euthanized 7 days postinjection. Various indicators of kidney and liver function were examined. These were creatinine, uric acid, blood urea nitrogen, albumin, total protein for kidney function, and enzymes aspartate transaminase (AST), alkaline phosphatase (ALP), alanine transaminase (ALT) for liver function. Blood samples were also collected for hematological analysis. The tested blood parameters include red blood cell (RBC), white blood cell (WBC), and platelet counts. The mice were euthanized 15 days, and the major organs (heart, liver, lung, kidney, and spleen) were harvested for histological analysis by H&E staining.

Statistical analysis

The data in this study are presented as mean ± standard deviation (SD). Statistical analysis was carried out using GraphPad Prism 9.0. Non-paired *t*-tests were employed for analysis of comparison between two groups, and one-way analysis of variance (ANOVA) was performed for comparison among multiple groups. Significance was defined as **p*<0.05, ***p*<0.01, ****p* < 0.001 and *****p* < 0.0001.

**Supplementary Figures S1-S19**

**Figure S1.** Illustration of thermoplasmonics for various applications.

**Figure S2**. Molecular structures of fluorophores as controls used in this work.

**Figure S3**. (A) Schematic diagrams of the preparation process of BHQ1-modified AuNRs, BHQ2-modified AuNSs and BHQ3-modified AuNPs. (B) Normalized UV-vis-NIR absorbance and Raman spectra of AuNRs and AuNR@sBHQ1 NPs in aqueous dispersion. (C) Normalized UV-vis absorbance and Raman spectra of AuNSs and AuNS@sBHQ2 NPs in aqueous dispersion. (D) Normalized UV-vis absorbance and Raman spectra of AuNPs and AuNP@sBHQ3 NPs in aqueous dispersion.

**Table S1.** Frontier orbital energies for BHQ1 and AuNR@sBHQ1 NPs

| **Contents** | **BHQ1** | **Au@sBHQ1 NPs** |
| --- | --- | --- |
| Band Gap (eV) | 0.6138 | 0.0249 |
| Eigenvalue of VBM (eV) | -4.269 | -2.4599 |
| Eigenvalue of CBM (eV) | -3.6551 | -2.435 |
| Fermi energy (eV) | -3.997 | -2.4361 |

**Figure S4**. Representative TEM images of AuNRs with different PDA shell thicknesses of 3, 6, 9, 11, 15 and 18 nm, obtained from dopamine concentrations of 0.05, 0.10, 0.15, 0.20, 0.25 and 0.30 mg/mL, respectively. Scale bar = 100 nm.

**Figure S5**. Normalized UV-Vis-NIR spectra of AuNRs coated with PDA from dopamine concentrations of 0.05, 0.10, 0.15, 0.20, 0.25 and 0.30 mg/mL, respectively.

**Figure S6**. Temperature changes of AuNR@mBHQ1 NPs with various concentrations after laser irradiation (1064 nm, 1.0 W/cm^2^) for 5 min.

**Figure S7**. (A) Representative TEM images of AuNSs with different PDA shell thicknesses of 2, 4, 6, 8, 10 and 12 nm. (B) Representative TEM images of AuNPs with different PDA shell thicknesses of 2, 4, 6, 8, 10 and 12 nm. Scale bar = 50 nm, 100 nm.

**Figure S8.** Normalized UV-Vis-NIR spectra of (A) AuNSs and (B) AuNPs coated with PDA from dopamine concentrations of 0.05, 0.10, 0.15, 0.20, 0.25 and 0.30 mg/mL, respectively.

**Figure S9.** PCE optimization of AuNS@mBHQ2 NPs by varying (A) dye-to-AuNS number ratio and (B) PDA shell thickness. The PDA shell thickness was fixed at ca. 4 nm in panel A. PCE optimization of AuNP@mBHQ3 elevate by varying (C) dye-to-AuNP number ratio and (D) PDA shell thickness. The PDA shell thickness was fixed at ca. 4 nm in panel C.

**Figure S10.** Heating-cooling curves and corresponding PCE of (A) AuNR@PDA, (B) AuNS@PDA and (C) AuNP@PDA NPs, excited by 1064, 785 and 633 nm, respectively. The PDA shell thickness in three NPs is ca. 6 nm.

**Figure S11**. (A) Heating-cooling curves, (B) cooling functions curve, and (C) corresponding PCE of AuNS@mIR783, AuNS@mICG and AuNS@mCy641 NPs. The average amount of confined reporter molecules per AuNS in three types of NPs in panel A-C is ca. 24000. (D) Heating-cooling curves, (E) cooling functions curve and (F) corresponding PCE of AuNP@mCV, AuNP@mNB and AuNP@mRh6G. NPs. The average amount of confined reporter molecules per AuNP in three types of NPs in panel D-F is ca. 18500.

**Table S2.** Calculation of packaged amounts of three BHQs inside PDA onto matched plasmonic nanostructures

| **Methods** | **Reporters** | **Theoretical value** | **Experimental value** |
| --- | --- | --- | --- |
| **SAMs** | **BHQ1** | 15215 | 12307 |
|  | **BHQ2** | 12604 | 10083 |
|  | **BHQ3** | 5652 | 4521 |
| **Multilayers** | **BHQ1** | / | 50089 |
|  | **BHQ2** | / | 28260 |
|  | **BHQ3** | / | 9075 |

Note that the experimental values for AuNR@mBHQ1, AuNS@mBHQ2, AuNP@mBHQ3 NPs with multilayer dyes were conducted on PDA thickness of ca. 11, 8 and 8 nm, respectively.

**Figure S12**. Aqueous stability of the hydrodynamic diameter of 1.0 nM AuNR@mBHQ1 NPs with different thickness of PDA during storage within 7 days.

**Figure S13**. Cellular viability of (A) 4T1 and (B) MCF-7 cells incubated with AuNR@mBHQ1 NPs at different concentrations for 24 and 48 h. Data are shown as means ± SD.

**Figure S14**. (A) Experimental setup of the deep-tissue photothermal test in chicken breast muscle tissues. (B) Statistical analysis was performed on the temperature changes of AuNR@mBHQ1 NPs covered with chicken breast meat of different thicknesses after irradiation with 808 nm and 1064 nm lasers for 5 min. The error bars were expressed as mean ± S. D. (n = 5). *p < 0.05, **p < 0.01, ***p < 0.001, and ****p < 0.0001

**Figure S15**. Body weight changes of the mice with five different treatments groups over 15 days.

**Figure S16**. Blood biochemical and hematology indices of the mice intravenously injected with PBS and AuNR@mBHQ1 NPs, respectively. Data represent mean ± S.D. from n = 3 mice per group. Abbreviations: aspartate aminotransferase (AST), creatinine (CREA), alanine aminotransferase (ALT), and blood urea nitrogen (BUN); red blood cell count (RBC), hemoglobin content (HGB), mean corpuscular hemoglobin concentration (MCHC), hematocrit (HCT), mean corpuscular hemoglobin (MCH), and mean corpuscular volume (MCV).

**Figure S17**. H&E staining of the main organs of mice after systemic administration of AuNR@mBHQ1 NPs for 15 days.

**Figure S18**. Quantitative biodistribution analysis of AuNR@mBHQ1 NPs in mice by measuring the Au content in the tumor and major organs at 6 h, 12 h, 7 d, and 28 d using ICP-MS.

**Figure S19**. (A) Time-dependent probe concentration profiles of the plasma of the mice after intravenous injection of AuNR@mBHQ1 NPs. (B) Cumulative Au excretion profiles of the feces from the mice.
